# Encapsulation of Fish Oil in Pullulan/Sodium Caseinate Nanofibers: Fabrication, Characterization, and Oxidative Stability

**DOI:** 10.3390/foods14213677

**Published:** 2025-10-28

**Authors:** Suaad Dabora, Bo Jiang, Khin Su Su Hlaing

**Affiliations:** 1 State Key Laboratory of Food Science and Resources, Jiangnan University, Wuxi 214122, China; suaddolly15@gmail.com (S.D.); khinsusuhlaing.ks@gmail.com (K.S.S.H.); 2 International Joint Laboratory on Food Safety, Jiangnan University, Wuxi 214122, China

**Keywords:** electrospinning, biopolymer, encapsulation, omega-3, molecular docking, DFT

## Abstract

This study aims to enhance the oxidative stability of fish oil through encapsulation in pullulan/sodium caseinate (PUL/NaCAS) nanofibers. Electrospinning was employed to produce three formulations: control (0% fish oil) and samples with 5% and 10% fish oil. Characterization of the emulsions showed that increasing oil content led to larger droplet size and reduced viscosity. Scanning electron microscopy (SEM) analysis revealed surface imperfections and a gradual increase in fiber diameter with higher oil loading. Fourier transform infrared (FTIR) spectroscopy confirmed molecular interactions, and fibers with 10% fish oil showed a shift toward a more amorphous structure. Fish oil incorporation also enhanced hydrophobicity and thermal stability, as indicated by thermal and wettability measurements. Antioxidant assays include 2,2-diphenyl-1-picrylhydrazyl (DPPH), 2,2′-azino-bis (3-ethylbenzothiazoline-6-sulfonic acid) (ABTS), and total phenolic content (TPC), which showed the highest bioactivity at 5% fish oil, with a slight decrease at 10%, likely due to structural saturation. Encapsulation at 5% fish oil significantly reduced lipid oxidation during storage (hydroperoxide values decreased from 8.6 to 4.8 mM at 60 °C/15 days), demonstrating the protective effect of the nanofiber matrix. Docking and density functional theory (DFT) analyses confirmed stable DHA/EPA–caseinate interactions and increased electronic stability, supporting the experimental results. Compared with conventional carriers such as spray-dried or maltodextrin-based systems, PUL/NaCAS nanofibers offered superior oxidative stability, bioactivity, and a biodegradable matrix. Overall, the 80PUL:20NaCAS:5% fish oil formulation represents a versatile platform for stabilizing omega-3 oils, with potential applications in food preservation, nutraceutical delivery, and functional packaging.

## 1. Introduction

In fish oil, omega-3 polyunsaturated fatty acids (PUFAs), including docosahexaenoic acid (DHA) and eicosapentaenoic acid (EPA), are vital bioactive molecules. Among the several health-promoting properties of these fatty acids, anti-inflammatory, cardioprotective, neurodevelopmental, and immunomodulating ones are most well-known. As such, their inclusion into nutritional supplements, functional foods, and pharmacological formulations has attracted a lot of interest [1]. Fish oil’s great sensitivity to oxidative deterioration makes its technological application still difficult, nevertheless. Heat, light, oxygen, and metal ions can start peroxidation, which results in rancid off-flavours, nutritional losses, and maybe dangerous secondary compounds [2].

Encapsulation has become a potential approach to guard delicate lipid molecules from environmental stresses in order to solve stability problems. Encapsulation increases shelf life, absorption, and dispersion in addition to oxidative stability [3]. Because electrospinning can generate nanofibrous materials with high surface area-to-volume ratios, porosity, and adjustable shape among the several encapsulating methods, it has become more and more widely used. These properties make electrospun fibers outstanding options for regulated bioactive protection and delivery [4]. Compared with conventional encapsulation methods such as spray-drying or nanoemulsions, electrospun pullulan/sodium caseinate nanofibers offer enhanced oxidative stability, controlled release, and improved bioactive retention due to their high surface area, fibrous structure, and tunable porosity, highlighting the novelty of this approach for stabilizing omega-3-rich oils [5].

Aureobasidium pullulans’ water-soluble exopolysaccharide, pullulan, is ideal for electrospinning because of its low oxygen permeability, film-forming capacity, and compatibility with food and pharmaceutical uses. The resultant matrix shows synergistic functioning when combined with sodium caseinate (NaCAS), a milk-derived amphiphilic protein with high emulsifying capabilities [6]. Pullulan gives structural integrity and protective encapsulation during fiber manufacture; sodium caseinate stabilizes emulsified fish oil droplets. Such biopolymer combinations have been found in past research to improve encapsulation efficiency and regulate lipophilic component leakage [7].

Though these benefits exist, little study has methodically examined how fish oil loading affects the structural, physicochemical, and oxidative stability of electrospun pullulan/sodium caseinate nanofibers [8]. With oil content, key factors like emulsion viscosity, fiber shape, surface wettability, crystallinity, thermal stability, and antioxidant activity may change greatly and hence influence encapsulation performance and product functioning [9]. This highlights the need for a systematic investigation to determine the optimal oil-to-polymer ratio and understand how oil loading impacts the overall stability and functionality of the nanofiber system.

Based on these considerations, we hypothesize that encapsulating fish oil in pullulan/sodium caseinate nanofibers via electrospinning will enhance oxidative stability and preserve bioactive properties, while modulating functional activities such as antioxidant and antidiabetic potential.

Thus, the objective of this work was to create and characterize pullulan and sodium caseinate nanofiber-based delivery systems for fish oil encapsulation. Three formulations—0%, 5%, and 10% fish oil—were made and examined in terms of emulsion stability, fiber shape, chemical structure, bioactivity, and oxidative stability. This work offers an insightful analysis of the design of nanostructured systems for the stability and delivery of omega-3-rich lipids in food and nutraceutical uses by clarifying the structure–function correlations and determining the ideal oil-to-polymer ratio.

This study presents a novel approach to stabilizing fish oil through its encapsulation in electrospun pullulan/sodium caseinate nanofibers. The novelty lies in combining pullulan’s excellent film-forming and oxygen-barrier properties with sodium caseinate’s strong emulsifying capacity, creating a synergistic carrier system that enhances oil entrapment and oxidative protection. Unlike previous studies, this work systematically evaluates the effect of varying oil loadings on fiber morphology, physicochemical stability, and bioactivity, thereby identifying the optimal formulation (5% fish oil) that preserves omega-3 fatty acids during storage. This distinct contribution provides both mechanistic understanding and practical insights for developing stable omega-3 delivery systems with potential applications in food preservation, nutraceutical delivery, and functional packaging.

## 2. Materials and Methods

### 2.1. Materials

Macklin Biochemical Co., Ltd., Shanghai, China, provided the fish oil derived from menhaden. NaCAS powder was acquired from Tokyo Chemical Industry Co., Ltd. (Kitaku, Tokyo, Japan), and pullulan (I.P. 20) was supplied by Beijing Yinokai Technology Co., Ltd. (Beijing, China).

#### Fish Oil Composition

The fish oil used in this study was derived from menhaden (Macklin Biochemical Co., Ltd., Shanghai, China). Its fatty acid profile was analyzed via gas chromatography (GC) and contained approximately EPA 18.5%, DHA 12.3%, palmitic acid 25.0%, oleic acid 10.2%, linoleic acid 4.5%, and other minor fatty acids (29.5%). This composition provides the necessary context for interpreting the oxidative stability and bioactive properties of the encapsulated nanofiber formulations.

### 2.2. Electrospinning Solutions Preparation

**Figure 1:**
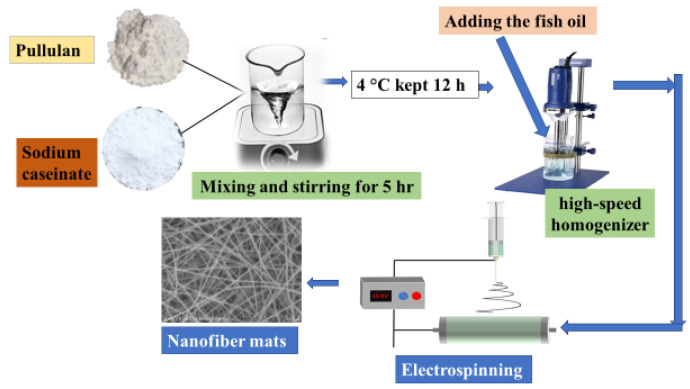
Schematic illustration of pullulan/sodium caseinate nanofiber preparation with fish oil.

PUL (80% *w*/*w* of polymer) and NaCAS (20% *w*/*w* of polymer) were dissolved in purified water to obtain a total polymer concentration of 20% *w*/*v*. Then the solution was stirred on a magnetic stirrer for 5 h at 25 °C and 500 rpm, and then left at 4 °C overnight to promote solubilization and allow all air to dissipate. After that, 5 g (moderate oil loading, 5% *w*/*w* relative to total polymer + oil) or 10 g (moderate oil loading, 10% *w*/*w* relative to total polymer + oil) of fish oil was added and homogenized using a high-speed homogenizer for 5 min at 15,000 rpm. The schematic representation of the nanofiber preparation process, including mixing, homogenization, and electrospinning steps, is shown in Figure 1. The fish oil concentrations (0%, 5%, and 10% *w*/*w*) were selected to represent control (without oil), moderate oil loading (5%), and high oil loading (10%); 5% was based on preliminary trials showing optimal encapsulation and fiber morphology, while 10% tested the upper limit to evaluate structural and functional impacts.

All formulations maintained the same polymer ratio (PUL/NaCAS = 80:20), with only the fish oil content varying. This setup ensures reproducibility, comparability across samples, and allows calculation of the emulsifier-to-oil ratio for each formulation, which is relevant to the discussion of interfacial coverage and droplet stability in Section 3.2.

### 2.3. Electrical Conductivity, Surface Tension, and pH

The electrical conductivity was achieved using the digital conductivity meter (Mettler Toledo, Greifensee, Switzerland). The surface tension was measured using an automatic surface tension meter (Powereach, Beijing, China). The pH was estimated using a digital pH meter (Hanna Instruments, Woonsocket, RI, USA) [10]. All measurements were performed in triplicate (n = 3).

### 2.4. Droplet Size

The droplet diameters of the emulsions were determined utilizing the NanoBrooker Omni Analyzer (Brookhaven Instruments, Holtsville, NY, USA), as described by Premi and Sharma [11], with modifications to sample dilution and temperature equilibration. To conduct the analysis, samples were warmed at 50 °C for 30 min to slightly reduce viscosity and ensure unrestricted Brownian motion of the droplets during measurement, followed by dilution and equilibration at 25 °C for 90 s. This controlled heating was used solely to facilitate accurate sizing without significantly affecting emulsion stability. The Sauter mean diameter (*D*3,2 nm) (defined as the diameter of a sphere with the same volume-to-surface area ratio as a particle of interest) was determined using Microsoft Excel employing Equation (1) as follows (where *n_i_* is the number of droplets of diameter *d_i_*):

(1) $$D3,2=\;\frac{\sum n_{i}d_{i}^{3}}{\sum n_{i}d_{i}^{2}}$$

The number-weighted average droplet diameter ($\bar{d}$; nm) was also calculated as follows:

(2) $$\bar{d}=\;\frac{\sum n_{i}dᵢ}{\sum n_{i}}$$

All droplet diameters are reported in nanometers (nm).

### 2.5. Viscosity

The viscosity of the emulsions was determined using the Mahdi, Al-Maqtari [12] method, with minor modifications. For each emulsion sample, three flow curves were obtained using an up–down–up shear rate protocol (1 → 300 → 1 → 300 s^−1^) to account for potential thixotropic behaviors. The upward curve (1 → 300 s^−1^) was used to calculate apparent viscosity. Measurements were estimated using controlled shear flow curves on a TA-Instruments rheometer (TA Instruments, HR-3, New Castle, DE, USA). The values were taken at 25 °C for 120 s, with shear rates that ranged from 1.0 to 300 s^−1^. Viscosity measurements were repeated three times (n = 3) for each emulsion sample.

### 2.6. Encapsulation Efficiency and Loading Capacity

LC and EE were calculated by measuring non-encapsulated fish oil [13]. Nanofiber (100 mg) was immersed in hexane (10 mL) for 1 min to remove the unencapsulated fish oil from the surface of the nanofibers. The mixture was filtered, and the fish oil’s absorbance was measured at 260 nm with a UV-vis spectrophotometer (Shimadzu UV-2600, Kyoto, Japan). The EE and LC values were computed using Equations (3) and (4):

(3) $$LC=\;\frac{Total\;theoretical\;mass\;of\;fish\;oil-Free\;mass\;of\;fish\;oil}{Mass\;of\;the\;nanofiber}$$

(4) $$EE=\frac{Total\;theoretical\;mass\;of\;fish\;oil-Free\;mass\;of\;fish\;oil}{Total\;theoretical\;mass\;of\;fish\;oil}$$

### 2.7. Electrospinning

The electrospinning method was carried out following Dabora, Jiang [6], with slight modifications to flow rate, voltage, and duration (Qingdao, China). The electrospinning equipment was employed with a high-voltage power supply set to 25 kV, at 25 ± 2 °C, and a relative humidity of 45 ± 5%. The anode was attached to a stainless-steel needle at 25 °C, which was coupled to a 10 mL disposable syringe containing the polymer solution. The syringe was mounted in a computer-controlled syringe pump in the middle of the collector, which consisted of a flat aluminum foil-covered plate with a 19-cm gap between the needle and the collector. The positively charged jet was created by the polymer solution, which slipped through the air gap and spread onto the collector at a flow rate of 0.75 mL/h. The electrospinning operation lasted 9 h.

### 2.8. Scanning Electron Microscopy (SEM)

SEM equipment (SU 1510, Hitachi Corp., Tokyo, Japan) was used to examine the morphological features of the nanofibers. The nanofiber samples were attached to a conductive adhesive and then coated with gold. The nanofiber samples were then scanned with an acceleration voltage of 5 kV and a magnification of 5.0 k. In the final step of this examination, the diameter distribution of the nanofibers was determined using ImageJ software (version 1.53t, National Institutes of Health, Bethesda, MD, USA) [4].

### 2.9. Water Contact Angle

To estimate the wettability of the nanofibers, the sessile drop method was used to measure the water contact angles of the nanofibers using a contact angle meter (DCAT-21, GmbH, Tübingen, Germany) and the SCA 20/21 programming protocol. A 5 μL of purified water was applied over the external surface of the nanofiber (2 cm × 2 cm), which was placed within a glass slide [14].

### 2.10. Fourier Transform Infrared Spectroscopy (FT-IR)

A NEXUS FTIR instrument (Nicholas Instruments Inc., Tucson, AZ, USA) equipped with an Attenuated Total Reflectance (ATR) Unit was used to study the chemical structure of the nanofibers. After blending the nanofiber samples with KBr powder, they were scanned in transmission mode with 32 scans at a resolution of 4 cm^−1^ and a wavenumber range of 500 to 4000 cm^−1^ [15].

### 2.11. Diffraction of X-Rays (XRD)

According to Wu, Zhong [16], a D2 PHASER X-ray diffractometer (BRUKER Co., Ltd., Karlsruhe, Baden-Württemberg, Germany) was used to measure the crystallinity of the nanofibers. It had a scanning range of 5° to 60° at a rate of 4° per minute and a 40 kV tube voltage.

### 2.12. Thermogravimetric Analysis (TGA)

To estimate the thermal stability of the nanofibers, the thermogravimetric analyzer (METTLER TOLEDO, Greifensee, Switzerland) was used to evaluate the thermogravimetry (TG). Approximately 5 mg of the nanofibers was placed in the crucible and heated from 25 to 650 °C at a heating rate of 20 °C under a nitrogen atmosphere [17].

### 2.13. Bioactive Properties

The bioactive properties of the nanofibers [Total phenolic content via Folin–Ciocalteu approach (TPC), Antioxidant activity by DPPH approach (DPPH), and Antioxidant capacity via ABTS approach (ABTS)] were determined as described by Al-Maqtari, Alkawry [18].

### 2.14. Antidiabetic Activity

The antidiabetic activity of the nanofibers was estimated based on α-amylase inhibition using the dinitrosalicylic acid (DNS) assay and α-glucosidase inhibition using p-nitrophenyl-β-glucopyranoside (pNPG) assay as described by Ali, Jiang [19]. α-Amylase and α-glucosidase inhibition assays were performed in triplicate (n = 3).

### 2.15. Oxidative Stability (Peroxide Values)

The peroxide value was determined to estimate the lipid oxidation in stored nanofibers at 4 °C, 25 °C, and 60 °C. The cumene hydroperoxide standard curve (0–20 μM) was used to calculate the lipid hydroperoxide concentrations as mM hydroperoxide/g [20]. All peroxide value measurements were performed in triplicate (n = 3) for each storage condition.

### 2.16. Molecular Docking and Density Functional Theory (DFT)-Based Simulation Procedures

In this meticulously crafted investigation, a robust fusion of traditional in silico methodologies—encompassing molecular optimization and precise, point-specific molecular docking—has been seamlessly integrated with cutting-edge density functional theory (DFT) simulation analyses. These sophisticated quantum mechanical explorations have been diligently applied to unravel the intricate properties of the compounds employed throughout this experimental endeavor.

#### 2.16.1. Molecular Identification and Optimization

A group of chemicals, including eicosapentaenoic acid (EPA, CID: 446284), docosahexaenoic acid (DHA, CID: 445580), and sodium caseinate (CID: 598), was retrieved from the PubChem database. The targeted macromolecule, casein, was retrieved from the RCSB PDB database [21,22]. All the optimized microcrystal ligands were saved as ‘mol2’ files, while the sole protein macromolecule was conserved as a ‘pdb’ file format. The protein active sites were identified using the COACH-D algorithm following standard methodology [23]. Besides, a sophisticated CAVER algorithm was utilized to figure out the best binding site of the protein, considering the protein tunnels embedded inside the α and β-helix structures [24].

#### 2.16.2. Molecular Docking and Post-Molecular Docking Analysis

A comprehensive point-specific supramolecular docking was conducted using PyRx 0.8 (AutoDock Vina, La Jolla, CA, USA), following standard methodology, where the binding affinity (kcal/mol) and noncovalent interactions such as hydrogen bonding and hydrophobic interactions were considered [25]. Following the molecular docking, the ligand–protein complex structures were observed in the PyMOL (Version 3.1) and BIOVIA (Version 4.5) Discovery Studio Visualizer tools sequentially [26]. As part of the post-molecular docking analysis, the quantitative measurement of the number of H-bonds, and the atomic distances of each of the hydrophobic amino acid residues from the EPA and DHA atoms were analyzed using the LigPlot+ (version 2.2) following established protocols, which works on Java Runtime Environment scripts [27]. Finally, the Molecular Mechanics Generalized Born Surface Area (MMGBSA) values for each of the ligand–protein complexes were analyzed.

#### 2.16.3. DFT Simulation Analysis

To analyze the chemical-to-chemical interactions based on the energy density matrix, a comprehensive DFT simulation for 200 ns was conducted individually for EPA and DHA with sodium caseinate. Parameters such as HOMO–LUMO band gap, self-consistent field potential (SCF), orbital-specific eigenvalues, Cartesian axial H-bond distribution with the corresponding energy fields, and the final atomic energy distribution considering the energy transition numbers over different simulation time frames were considered [28]. To execute the whole DFT simulation, Gaussian 16 and ArgusLab simulators were used based on standard procedures [29,30].

### 2.17. Statistical Analysis

The triplicate data (n = 3) of the results were employed to ensure reproducibility. The data were statistically analyzed with IBM SPSS 20 software (SPSS Inc., Chicago, IL, USA). A one-way analysis of variance (ANOVA) was used to identify significant differences with a *p*-value of ≤0.05. The figures were created with Origin 2018 (OriginLab, Northampton, MA, USA). All the statistical analyses of the complicated data obtained from the DFT analysis were conducted using the ‘R programming language’ and ‘GraphPad Prism 8.0.1’ combined to ensure maximum accuracy [31,32].

## 3. Results and Discussion

All data are presented as mean ± SD with the number of replicates indicated for each measurement (n = 3). Figures include error bars and clear statistical annotations to highlight significant differences (*p* < 0.05), and figure captions have been revised for clarity, accurate sample labeling, and to reflect the scope of each analysis.

### 3.1. Emulsion Characterizations

#### 3.1.1. Electrical Conductivity, Surface Tension, and pH

**Figure 2:**
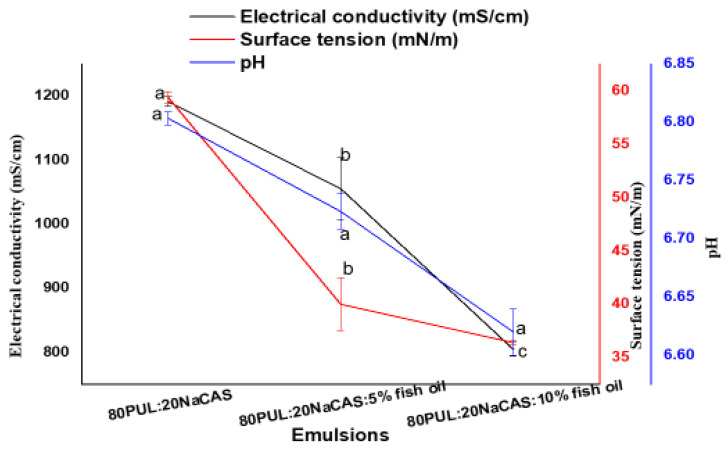
Electrical conductivity, surface tension, and pH of PUL/NaCAS emulsions with varying fish oil contents. Data are presented as mean ± SD (n = 3). Different letters indicate significant differences (*p* < 0.05); pH differences were not significant (*p* > 0.05).

Figure 2 shows how fish oil inclusion affects pullulan/sodium caseinate emulsions’ electrical conductivity, surface tension, and pH. Understanding emulsion stability and its fit for electrospinning uses depends on these criteria.

The fish oil content greatly reduced the electrical conductivity. The maximum conductivity (~1200 ± 15 mS/cm^a), which dropped to ~1050 ± 12 mS/cm^b and ~800 ± 10 mS/cm^c in the 5% and 10% fish oil formulations, respectively (Figure 2), was shown by the control formulation (80PUL:20NaCAS). The growing fraction of non-polar oil droplets, which dilute the continuous aqueous phase and impede ionic mobility, can help to explain this drop [33]. Furthermore, influencing ion availability could be the polymer matrix’s encapsulation of ions and potential interactions with sodium caseinate [7].

Comparably, surface tension dropped significantly from over 59 ± 0.6 mN/m^a in the control to almost 36 ± 0.2 mN/m^c in the 10% fish oil emulsion (Figure 2). This is probably related to sodium caseinate’s amphiphilic character, which lowers interfacial tension by adsorbing at the oil–water contact [34]. Generally speaking, lower surface tension is good for electrospinning since it helps steady jets and uniform fibers to develop [35].

From ~6.81 ± 0.01^a in the control to ~6.62 ± 0.02^c in the 10% fish oil sample (Figure 2). This slight acidification is likely due to the free fatty acids already present in the fish oil, with higher oil concentrations resulting in a greater total free fatty acid content. The partial hydrolysis of fish oil during emulsification or small oxidative events, producing free fatty acids, could help to explain this tendency by somewhat acidifying the media. Furthermore, protein–lipid interactions might change the system’s ionization equilibrium [36].

The noted variations in pH, surface tension, and electrical conductivity generally reflect the increasing effect of fish oil inclusion on emulsion characteristics. These changes should affect oxidative stability, encapsulation efficiency, and fiber generation, as discussed in the next sections.

The observed pH changes of approximately 0.2 units were not statistically significant (*p* > 0.05), indicating minimal effect of fish oil incorporation on the solution pH.

#### 3.1.2. Droplet Size

**Figure 3:**
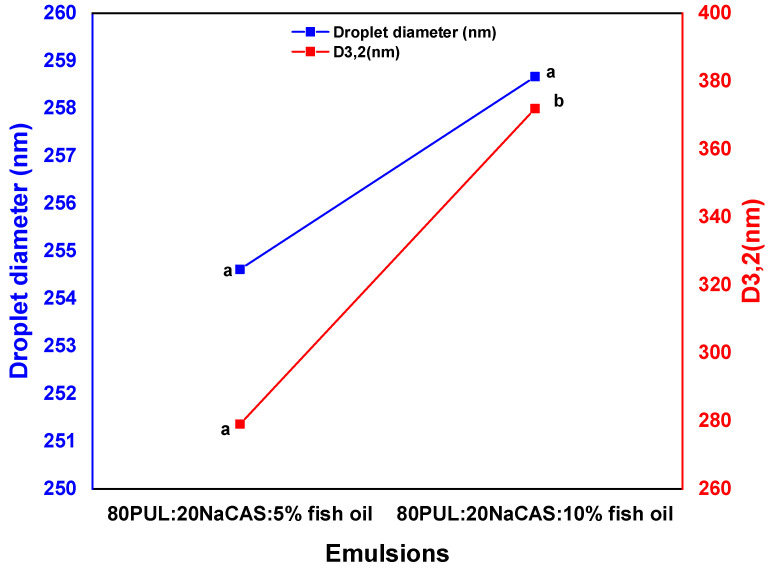
Effect of fish oil content on emulsion droplet size. Data are presented as mean ± SD (n = 3). Different letters indicate significant differences (*p* < 0.05).

Figure 3 shows how fish oil incorporation affects the mean droplet diameter and surface-weighted mean diameter (D_3,2_) of the emulsions. Fish oil made both parameters rise gradually.

For the oil-containing emulsions, the droplet size increased to ~254 ± 10 nm^b and ~258 ± 9 nm^b in the emulsions that included 5% and 10% fish oil, respectively. Likewise, with 5% and 10% fish oil, the D_3,2_ values rose from ~279 ± 10 nm^a at 5% oil to ~371 ± 12 nm^b at 10% oil. The control sample (0% oil) was excluded from this analysis, as no oil droplets are present, and droplet size cannot be measured. The larger oil content expected by the increase in droplet size encourages droplet coalescence or aggregation during emulsification, especially at higher concentrations where the emulsifying capacity of the system may become saturated [33].

These findings show that although a good emulsifier, sodium caseinate, can have a limited capacity to maintain larger amounts of oil within the given polymer ratio [37]. As in the 10% fish oil formulation, an excessively low emulsifier-to-oil ratio results in insufficient interfacial coverage, which produces bigger, less stable droplets [38].

Larger droplets directly reduce emulsion stability because the available surface area for emulsifier adsorption decreases, making complete interfacial coverage difficult. This facilitates coalescence, aggregation, and potential phase separation over time [39].

During electrospinning, larger droplets can disrupt the polymer jet, leading to bead formation, irregular fiber diameters, or localized ruptures, while smaller, well-stabilized droplets integrate more uniformly, producing smooth, continuous fibers with consistent morphology [40].

As it can cause phase separation, oil leakage, and fiber flaws, an increase in droplet size can negatively affect emulsion stability and the quality of the electrospun nanofibers. Thus, guaranteeing successful encapsulation and long-term stability depends on reducing droplet size by ideal emulsifier/oil ratios [41].

#### 3.1.3. Viscosity

**Figure 4:**
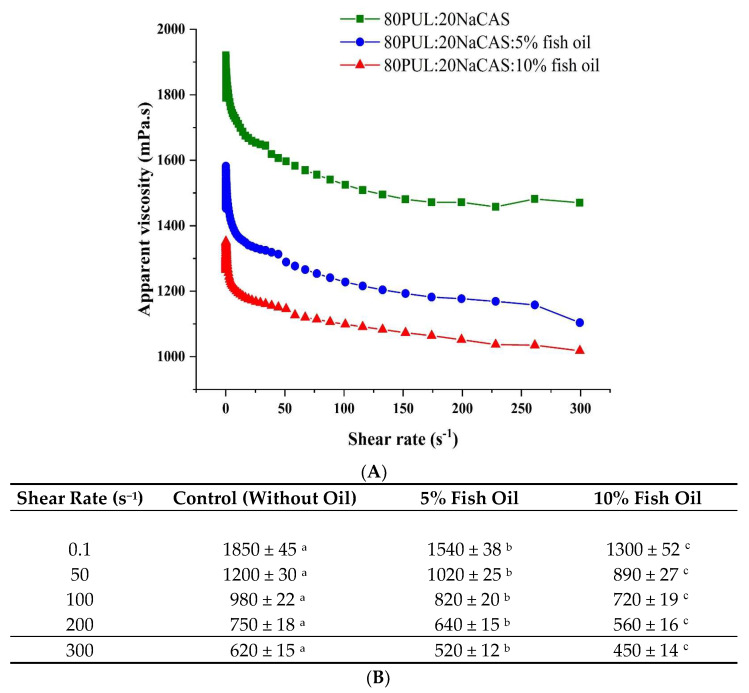
(**A**) Apparent viscosity of the emulsions. (**B**) Apparent viscosity values (mPa·s) of PUL/NaCAS emulsions at selected shear rates. Data are mean ± SD (n = 3). Different letters within the same row indicate significant differences (*p* < 0.05).

Figure 4 shows the apparent viscosity profiles of the pullulan/sodium caseinate emulsions at various fish oil concentrations. Typical for polymeric and protein-based emulsions, all emulsions showed shear-thinning behavior, defined by a reduction in viscosity with increasing shear rate [42].

Starting at about 1850 mPa·s and progressively lowering with increasing shear, the control emulsion (80PUL:20NaCAS) showed the maximum viscosity across all shear rates. Starting at about 1540 mPa·s and 1300 mPa·s, respectively, the emulsions containing 5% and 10% fish oil showed gradually decreased viscosities. There can be various reasons for this drop in viscosity with rising oil content.

First, fish oil adds a distributed hydrophobic phase that can upset the continuous polymer matrix, thereby lowering bulk viscosity and maybe causing less chain entanglement [43]. Second, the emulsion system becomes more heterogeneous and less cohesive as fish oil concentration rises since sodium caseinate has limited power to stabilize greater oil quantities [44]. Particularly, pullulan, which lowered intermolecular interactions across polymer chains, leads to lower resistance to flow [45].

In processing, particularly during electrospinning, lower viscosity at higher oil concentrations may be beneficial because it facilitates easier jet production. To maximize encapsulation and fiber shape, however, too low viscosity could damage fiber uniformity and structural integrity, suggesting the requirement of balancing oil loading with matrix viscosity [9].

The complementary functions of sodium caseinate and pullulan help to explain these viscosity and stability trends. Sodium caseinate, as an amphiphilic protein, primarily acts at the oil–water interface by reducing interfacial tension and stabilizing lipid droplets through electrostatic and steric effects [46,47]. Conversely, pullulan contributes to bulk viscosity and forms an oxygen-impermeable matrix; its excellent film-forming capacity and low oxygen permeability are well documented [48], and higher molecular-weight pullulan enhances these barrier properties even further [49]. Together, NaCAS ensures efficient droplet stabilization while pullulan reinforces structural integrity and oxidative protection.

### 3.2. Nanofiber Characterizations

#### 3.2.1. Encapsulation Efficiency and Loading Capacity

**Figure 5:**
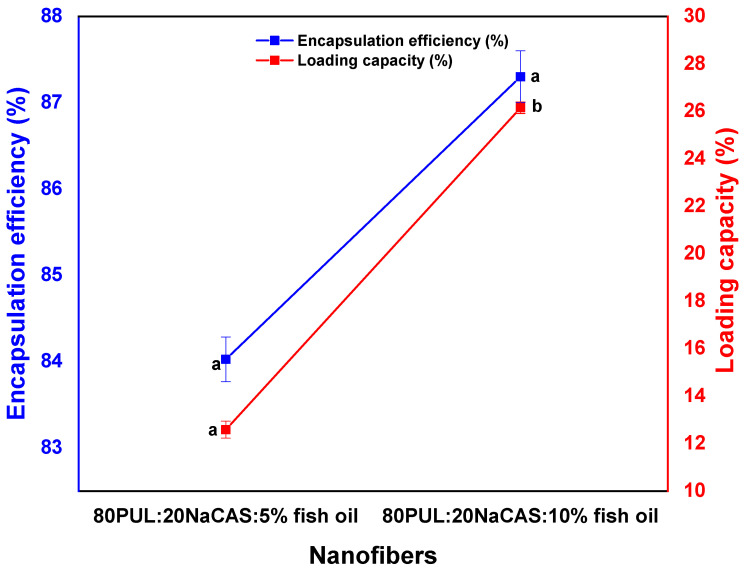
Encapsulation efficiency (EE) and loading capacity (LC) of PUL/NaCAS nanofibers with different fish oil contents. Data are presented as mean ± SD (n = 3). Different letters indicate significant differences (*p* < 0.05).

Figure 5 shows the loading capacity (LC) and encapsulation efficiency (EE) of fish oil within pullulan/sodium caseinate nanofibers. Higher fish oil concentration in the electrospinning fluid raised both values.

The encapsulation efficiency for the emulsion, including 5% fish oil, was almost 84.02 ± 0.24%^a (Figure 5); for the 10% fish oil formulation, it rose to 87.30 ± 0.3%^b (Figure 5). Likewise, as the starting oil content climbed, the loading capacity changed from 12.6 ± 0.35%^a to 26.2 ± 0.24%^b (Figure 5), suggesting a higher quantity of fish oil kept within the nanofiber matrix.

The high EE values (over 80%) across both formulations point to an efficient pullulan/sodium caseinate matrix in entrapping the lipophilic fish oil during electrospinning. Higher oil loading increases LC and EE; this is ascribed to improved availability of core material for entrapment and maybe improved droplet–matrix integration during fiber production [50].

A moderate viscosity reduction observed with increasing fish oil content also contributed to this effect by facilitating smoother jet initiation during electrospinning. Nevertheless, if viscosity decreases below critical thresholds (reported at ~500–800 mPa·s for polysaccharide–protein systems), insufficient chain entanglement may lead to bead formation, fiber breakage, or poor encapsulation [35]. Since all emulsions in this study remained well above this threshold, fiber continuity and uniformity were preserved. However, beyond the tested 10% oil loading, excessive thinning may destabilize the process, suggesting a practical upper boundary for maintaining electrospinnability and high encapsulation efficiency.

Still, a balance has to be kept since too high oil content could cause fiber flaws or oil leaks from polymer matrix saturation or decreased fiber integrity [51]. These findings suggest that pullulan/sodium caseinate nanofibers have enormous potential to encapsulate bioactive oils and thereby protect sensitive molecules like fish oil from environmental damage.

These results indicate that while 5% fish oil formulation achieves optimal encapsulation, the 10% formulation exhibits reduced performance. The larger droplet sizes and lower viscosity at higher oil content contribute to interfacial instability during electrospinning, promoting partial phase separation and heterogeneous fiber formation. Consequently, some oil may migrate to the fiber surface, generating defects and limiting accessibility of bioactive compounds. This saturation effect of the polymer matrix likely explains the observed decrease in antioxidant activity and oxidative protection at 10% oil. Such behavior aligns with prior studies demonstrating that excessive oil loading can destabilize protein–polysaccharide systems, reduce structural integrity, and compromise functional performance [52].

#### 3.2.2. Morphological Features by SEM

**Figure 6:**
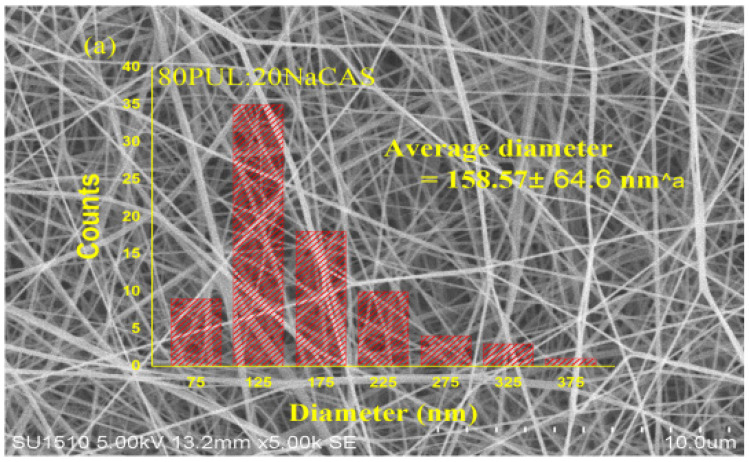

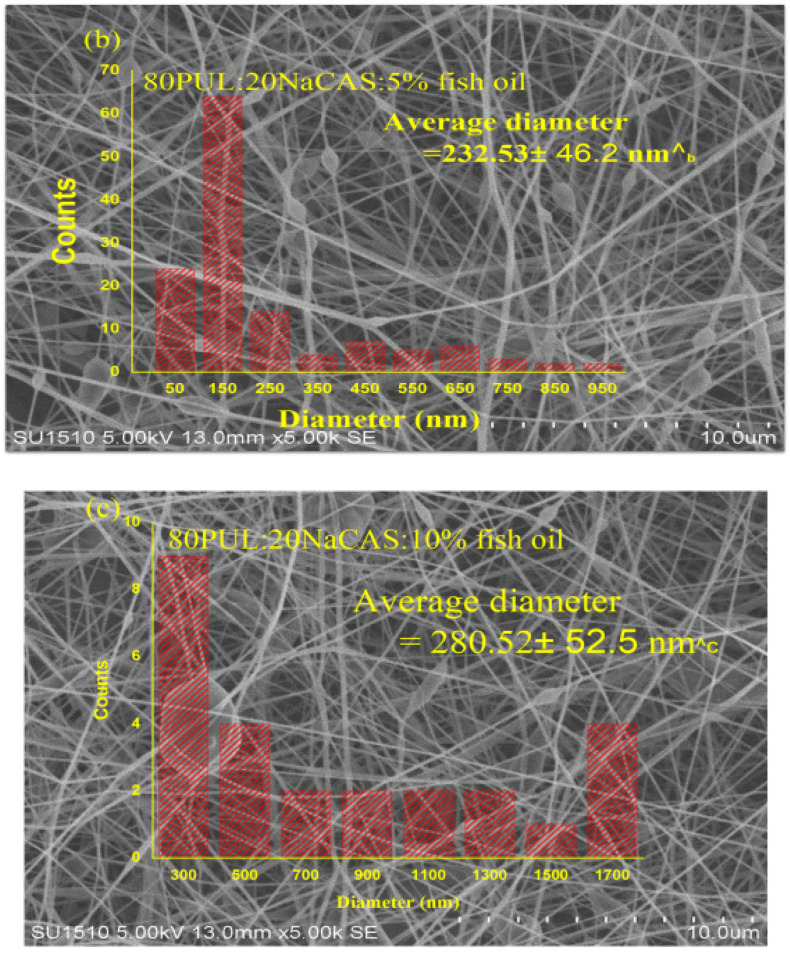
SEM images and diameter distribution of PUL/NaCAS nanofibers with (**a**) 0%, (**b**) 5%, and (**c**) 10% fish oil. Data are presented as mean ± SD (n = 50 fibers). Different letters indicate significant differences (*p* < 0.05).

As the amount of fish oil increased in the pullulan/sodium caseinate mix, scanning electron microscopy showed a noticeable change in the shape of the fibers (Figure 6). The control fibers, which had a consistent mix of polymers and stable electrospinning conditions, were smooth and uniform, with an average diameter of about 159 ± 64.6 nm. However, when 5% fish oil was added, the size of the fibers varied more, and the average diameter increased to around 233 ± 46.2 nm, with some thicker areas or small bead-like formations. The control fibers were smooth and even, with most of them around 159 nm in diameter, showing that the polymers mixed well and the electrospinning process was stable. On the other hand, adding 5% fish oil expanded the size distribution and raised the average fiber diameter to ~233 nm with sporadic thicker sections or minor bead-like development. At 10% oil loading, the fibers became much thicker and less uniform, with some flattened shapes and areas that likely formed because the hydrophobic oil did not mix well with the water-based polymer, resulting in an average diameter of about 281 ± 52.5 nm and sizes ranging from 300 to over 1700 nm. Although the average droplet size in the emulsion (~371 ± 12 nm) was larger than the mean fiber diameter, this apparent discrepancy can be explained by droplet breakup, elongation, and deformation during the electrospinning process, where strong shear and electric field forces reduce droplet size before or during fiber solidification [53].

Important functional consequences follow from these structural changes. The size and uniformity of the fibers affect how well they can trap substances and protect against outside elements in electrospun systems; thinner and more uniform fibers typically trap more effectively and offer better protection from oxidation because they cover the contents more completely and expose less lipid to oxygen [54].

The observed changes in fiber morphology with increasing fish oil content have direct implications for encapsulation efficiency and oxidative stability. Pullulan/sodium caseinate nanofibers with lower oil concentrations (0–5%) exhibited thinner and more uniform fibers, providing consistent polymer coverage that efficiently entrapped the oil and minimized its exposure to oxygen [54]. Increasing the fish oil content to 10% led to thicker fibers with greater heterogeneity, including occasional bead formation and aggregation, which slightly reduced localized oxidative protection but still maintained high overall encapsulation efficiency (~88%) [55]. These findings demonstrate a trade-off between maximizing oil loading and maintaining fiber uniformity, in agreement with previous studies on hydrophobic oil incorporation into electrospun fibers [56].

Pullulan nanofibers containing 10% fish oil had a high encapsulation efficiency of about 88%, as reported by García-Moreno, Damberg [57], which led to better protection against oxidation, especially when natural antioxidants were added. Conversely, the researchers found that excessively high oil levels (30 weight-based %) resulted in increased bead formation, which ironically correlated with improved oxidative stability.

These findings are consistent with studies on camellia oil–loaded zein fibers, where increasing the oil concentration led to a significant increase in nanofiber diameter [56]. Increasing the fish oil level to 10% dramatically changes fiber generation. The higher amount of oil likely disrupts how well the polymer can be spun into fibers, which results in less uniform fibers and increases the chance of creating beads or clumps of fiber [9]. Although caseinate can improve the miscibility of fish oil in the aqueous matrix, incomplete disruption of larger oil droplets during electrospinning may result in their aggregation or release onto the nanofiber surface, contributing to irregular fiber morphology [57].

#### 3.2.3. Wettability of the Nanofibers by Water Contact Angle

**Figure 7:**
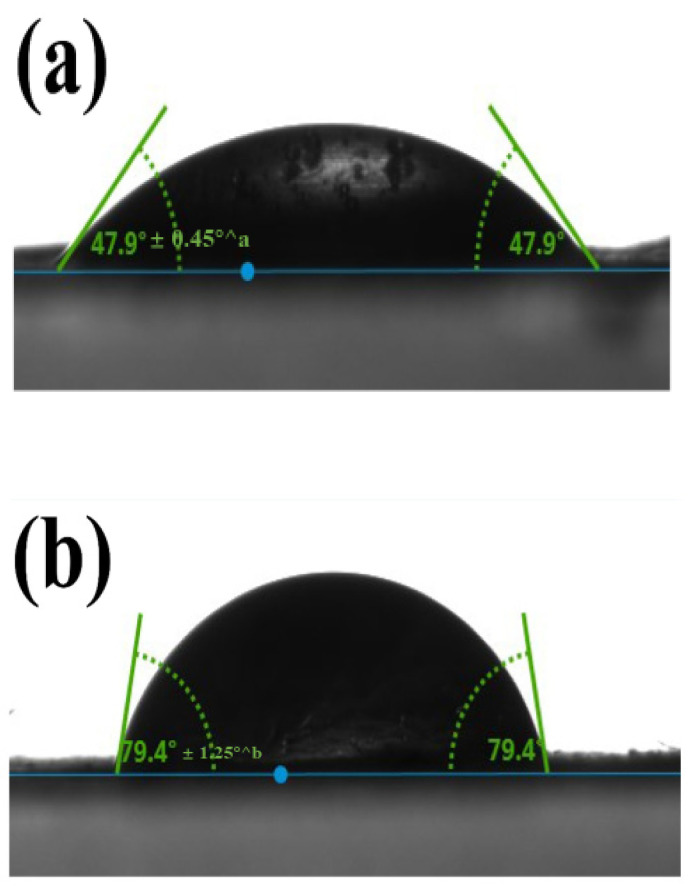

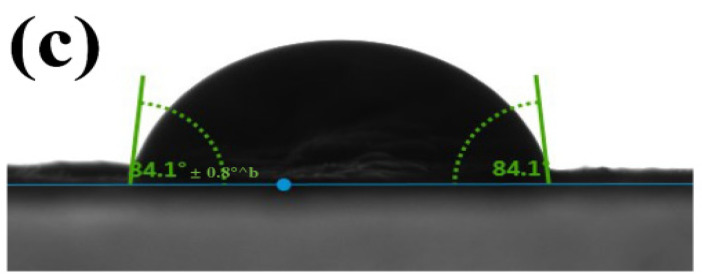
Water contact angle of PUL/NaCAS nanofibers with different fish oil contents: (**a**) 0%, (**b**) 5%, and (**c**) 10% fish oil. Data are presented as mean ± SD (n = 3). Different letters indicate significant differences (*p* < 0.05).

An important factor affecting the barrier characteristics, interaction with encapsulated compounds, and general oxidative stability of integrated bioactives of nanofibers is their surface wettability. Using water contact angle (WCA) measurements, the hydrophilicity—that is, hydrophobicity—of the pullulan/sodium caseinate nanofiber mats with increasing fish oil content was assessed. With a rather low contact angle of 47.9° ± 0.45°^a, the control illustrated in Figure 7a reflects a highly hydrophilic surface (Figure 7a). The contact angle rose to 79.4° ± 1.25°^b (Figure 7b) with the addition of 5% fish oil; an additional increase in fish oil to 10 g produced a contact angle of 84.1°± 0.8°^b (Figure 7c), hence demonstrating increased surface hydrophobicity.

The increasing WCA with fish oil loading points to a successful integration of the hydrophobic lipid phase within the nanofiber matrix, hence enhancing the surface with non-polar moieties. This trend corresponds with results of earlier investigations showing a change toward hydrophobic behavior upon encapsulation of lipophilic chemicals in hydrophilic biopolymer matrices [58]. Improving the oxidative stability of fish oil depends especially on such a change in surface qualities since a more hydrophobic surface might act as a barrier to ambient moisture and oxygen, therefore reducing oxidative degradation processes [59].

Additionally, being more hydrophobic may help keep fish oil droplets better contained within the nanofiber network, which reduces their exposure to factors that can cause oxidation. Previous studies confirming this effect have linked the inclusion of fish oil in electrospun matrices to both higher contact angles and lower peroxide values during storage [60]. Thus, the observed increase in WCA across the three formulations points to a dual function for fish oil: as a functional bioactive and as a structural modification improving the protective performance of the nanofiber carrier system.

#### 3.2.4. Chemical Structure by FTIR

**Figure 8:**
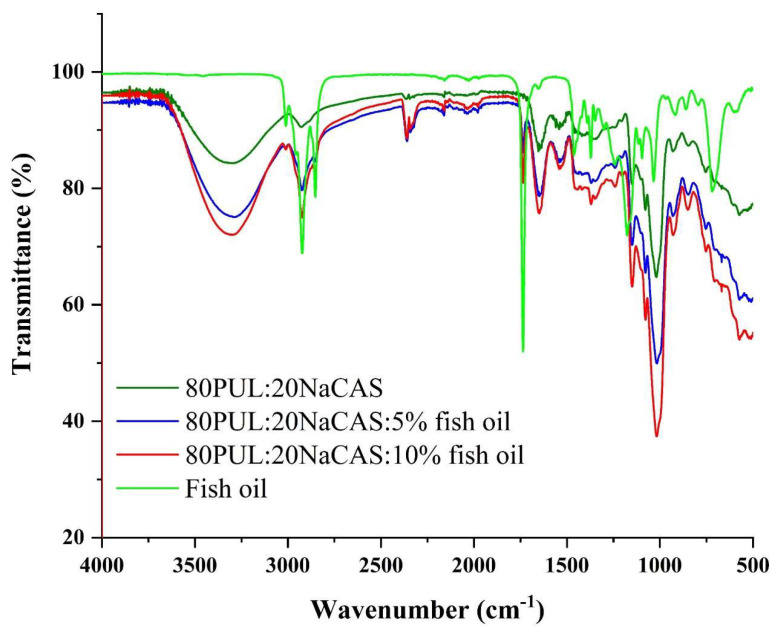
FTIR spectra of pullulan/sodium caseinate nanofibers with varying fish oil content.

Chemical interactions and structural changes within the pullulan/sodium caseinate (PUL/SC) nanofiber matrices upon fish oil incorporation were investigated using Fourier-transform infrared (FTIR) spectroscopy. As shown in Figure 8, the spectra of the blank nanofiber formulation (0 g fish oil) displayed characteristic bands for polysaccharides and proteins, including broad O–H stretching at 3300 cm^−1^, C–H stretching near 2925 cm^−1^, and amide I and II bands at 1650 cm^−1^ and 1540 cm^−1^, respectively. These values agree with other studies on PUL/SC-based systems [61].

Fish oil additions of 5% and 10% (*w*/*w*) were seen to cause significant spectrum alterations. Especially, fish oil loading raised the strength of the C–H stretching vibrations at 2925 cm^−1^ and 2855 cm^−1^, therefore verifying the existence of long-chain aliphatic groups from triglycerides [62]. Furthermore, the widening and minor displacement of the O–H band about 3300 cm^−1^ points to hydrogen bonding interactions between hydroxyl groups in pullulan and polar groups in fish oil or protein components, therefore helping to stabilize the encapsulated oil inside the matrix [63].

Further evidence for the effective integration of fish oil comes from the development and strengthening of bands in the range 1740–1750 cm^−1^, which match C=O stretching in ester groups. Especially noticeable in the 10% fish oil-loaded nanofibers, this band closely mimics the spectral profile of pure fish oil. These results show strong physical compatibility and possible chemical interactions among the fish oil, pullulan, and sodium caseinate, most likely by means of hydrogen bonding and hydrophobic interactions [64].

Such interactions help to increase the oxidative stability of fish oil that has been encapsulated. Intermolecular hydrogen bonding and encapsulation within a biopolymeric matrix can limit oxygen movement and prevent pro-oxidant access, therefore slowing lipid peroxidation reactions [65]. Moreover, the amphiphilic character of sodium caseinate helps oil droplets within the fiber network to be emulsified and stabilized, therefore lowering the possibility of oil leakage and oxidative stress [7]. All things considered, the FTIR data demonstrate effective encapsulation of fish oil and expose molecular-level proof for the protective interactions supporting better oxidative stability.

#### 3.2.5. Crystallinity by XRD

**Figure 9:**
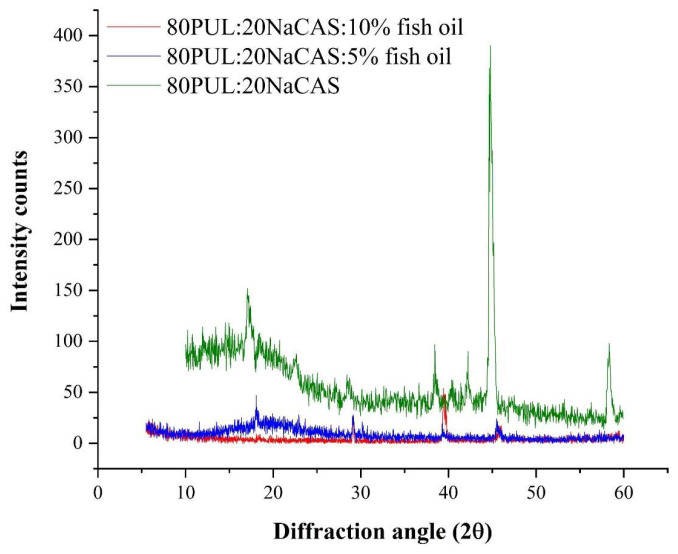
X-ray diffraction analysis of the nanofibers.

The structural organization and crystallinity of pullulan/sodium caseinate nanofibers with and without fish oil incorporation were evaluated using X-ray diffraction (XRD) analysis. Figure 9 shows the XRD pattern of the control sample, which clearly shows a crystalline peak around 2θ = 44° together with more general diffraction halos between 15° and 25°, therefore reflecting the semi-crystalline character of the biopolymer matrix. This pattern is compatible with earlier published XRD profiles of pullulan-based materials, which usually show poor crystallinity because of the amorphous structure of pullulan and partial ordering introduced by sodium caseinate [6].

Particularly in the area about 44°, a significant decrease in peak intensity and sharpness was noted when 5% and 10% fish oil were used, implying a disturbance in the molecular ordering and a move toward a more amorphous structure. The integration of the lipid phase into the nanofiber matrix most likely causes the reduced crystallinity since it disturbs the normal packing of the polymer chains and intermolecular interactions [66]. Most likely acting as plasticizers, the fish oil droplets cause disturbances in hydrogen bonding and crystalline alignment in the biopolymeric network [67].

One could argue that this lower crystallinity helps to maintain oxidative stability. Because they lack rigid structural domains that would allow microchannel development, amorphous matrices can offer superior encapsulation efficiency and operate as more effective diffusion barriers to oxygen and pro-oxidants than their crystalline counterparts [68]. Moreover, the more homogenous distribution of fish oil made possible by the higher molecular mobility and flexibility of the amorphous matrix can help to lower localized concentrations sensitive to oxidation [69]. Comparable decreases in crystallinity have been reported for oil-loaded polysaccharide/protein fibers, where the lipid phase acts as a plasticizer, disrupting molecular packing, similar to our observations [66]. These results align with earlier pullulan-based studies reporting semi-crystalline matrices with limited ordering due to sodium caseinate [6].

#### 3.2.6. Thermal Stability

**Figure 10:**
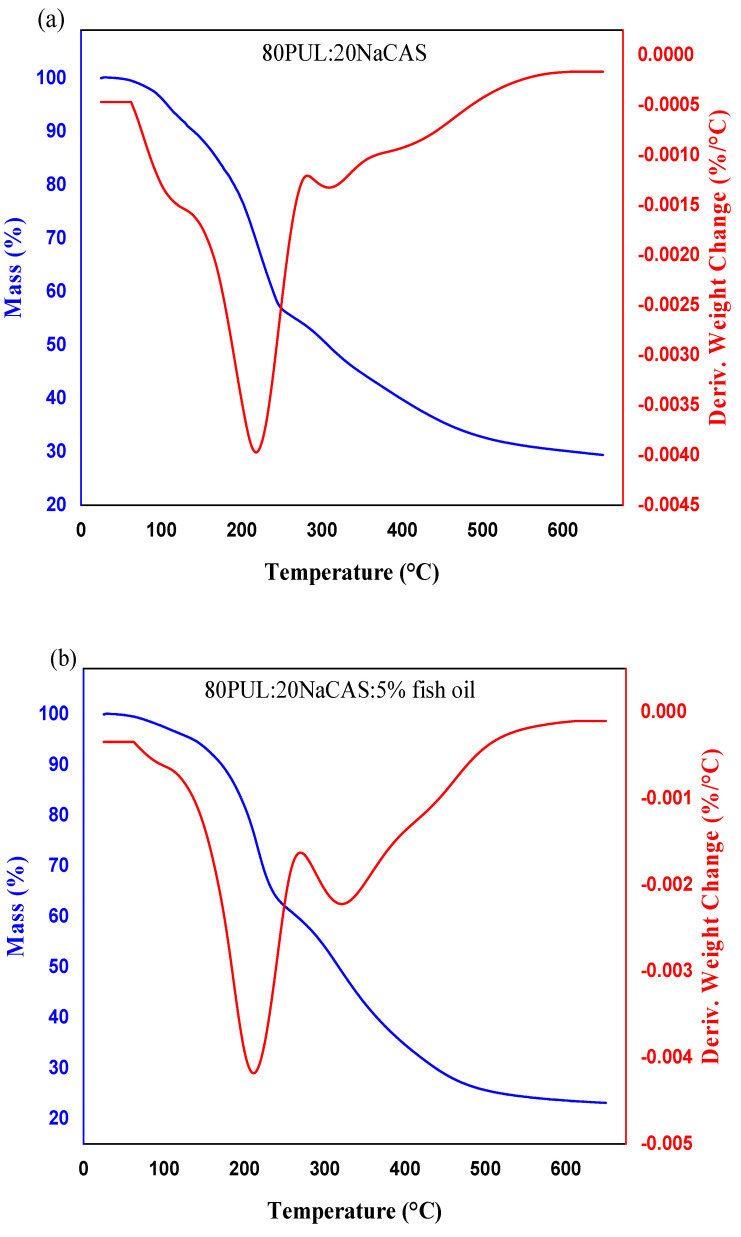

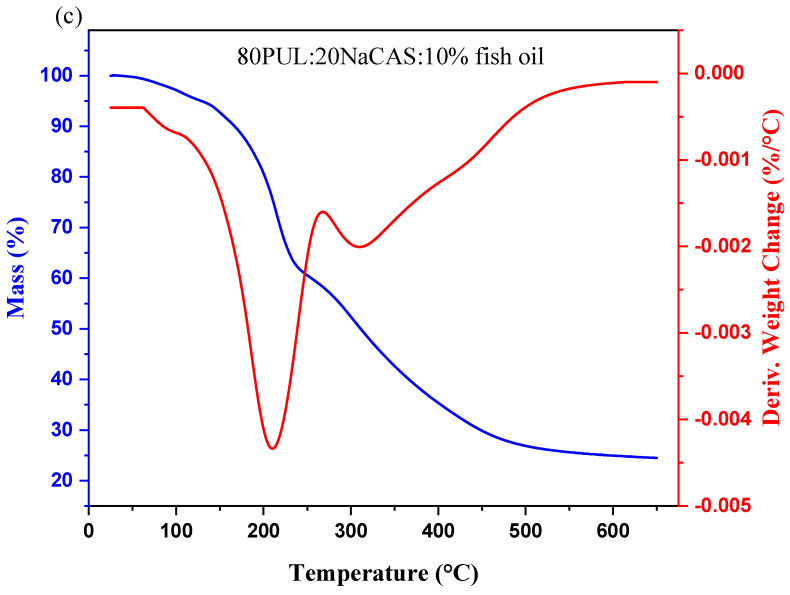
Thermal stability of pullulan/sodium caseinate nanofibers with varying fish oil content (**a**) 0%, (**b**) 5%, and (**c**) 10% fish oil, assessed by TGA (weight loss) and DTG (derivative weight loss). Curves illustrate the mass loss and degradation rate as a function of temperature. Data are presented as mean ± SD (n = 3), and error bars represent standard deviation.

Using thermogravimetric analysis (TGA) and derivative thermogravimetry (DTG), the thermal stability of the pullulan/sodium caseinate nanofiber compositions, both with and without fish oil, was assessed. For the three formulations, the mass loss profiles and corresponding breakdown rates are shown in Figure 10 by the blue TGA curves and red DTG curves.

The control nanofiber had a usual two-step deterioration pattern. The first weight loss below 150 °C is related to moisture evaporation and loss of weakly bound water. Attributed mostly to the breaking of glycosidic and peptide linkages, the second major degradation stage took place between 250 and 400 °C and resulted from the thermal breakdown of the polysaccharide–protein matrix [3]. This structural breakdown is supported by the occurrence of a clear DTG peak at about 300 °C [70]. Fish oil added produced changes in thermal behavior. The start of significant thermal degradation was somewhat delayed in both the 5% and 10% fish oil samples, suggesting a thermal stability enhancement, consistent with previous reports on oil–protein–polysaccharide interactions reducing molecular mobility and enhancing resistance to thermal stress [71]. With a downward shift in peak intensity, the 10% fish oil-loaded nanofibers displayed a larger DTG peak, implying a slower degradation process. The plasticizing impact of the oil droplets and the hydrophobic character of fish oil help to explain this improved stability by perhaps contributing to greater matrix–oil interactions, hence increasing the energy needed to start thermal decomposition [13].

Other studies involving oil encapsulation within protein–polysaccharide matrices have also shown similar thermal stabilization effects where oil–polymer compatibility and the formation of secondary interactions (e.g., hydrogen bonding, hydrophobic interactions) significantly lower molecular mobility and increase resistance to thermal and oxidative stress [72].

#### 3.2.7. Bioactive Properties

**Figure 11:**
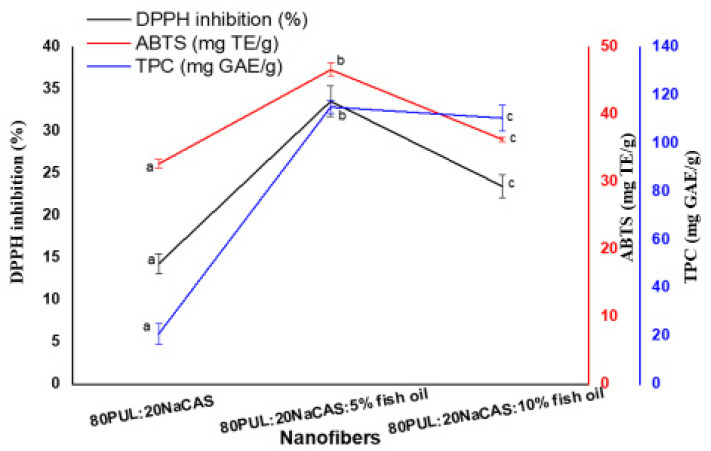
Bioactive properties of PUL/NaCAS nanofibers with different fish oil contents. Data are presented as mean ± SD (n = 3). Different letters indicate significant differences (*p* < 0.05).

Three methods—DPPH radical scavenging activity, ABTS radical cation decolorization, and total phenolic content (TPC)—were used to characterize the antioxidant properties and phenolic content of the nanofibers with and without encapsulated fish oil (Figure 11). With DPPH inhibition of about 14% ± 4.12 ^a, ABTS capacity of roughly 32 ± 0.63 mg TE/g ^a, and TPC of almost 21 ± 0.16 mg GAE/g ^a, the control nanofibers showed the lowest antioxidant activity. All three measurements of antioxidant activity rose dramatically upon addition of 5% fish oil; DPPH inhibition reached ~33% ±3.16 ^b, ABTS climbed to 46 ± 0.94 mg TE/g ^b, and TPC peaked at 115 ± 0.84 mg GAE/g ^b.

Natural antioxidants such as tocopherols and polyunsaturated fatty acids in fish oil are responsible for this increase in bioactivity; it should be noted that the observed antioxidant activity arises from these intrinsic compounds rather than the omega-3 fatty acids (EPA/DHA) themselves, and they may interact synergistically with the encapsulating biopolymer matrix to increase radical scavenging efficacy [73]. Fascinatingly, a further increase to 10% fish oil produced a modest drop across all three indicators—especially DPPH inhibition, which dropped to ~23% ± 2.31 ^c; ABTS dropped to 36 ± 0.36 mg TE/g ^c, and TPC dropped to 110 ± 0.27 mg GAE/g ^c. Beyond an ideal loading level, this tendency indicates that probable phase separation, aggregation of oil droplets, or polymer matrix saturation may weaken the antioxidant activity, therefore impeding the diffusion and accessibility of bioactive chemicals [52].

These findings align with earlier research demonstrating that while excess oil might destabilize the nanostructure and lower efficiency, moderate quantities of encapsulated lipophilic antioxidants boost bioactivity [74]. Moreover, the improved antioxidant activity at 5% fish oil supports the oxidative stability results from thermal and structural studies (Section 3.2.7, Section 3.2.8 and Section 3.2.9), thus indicating that the 80PUL:20NaCAS:5% fish oil formulation provides an ideal mix between structural integrity, thermal resistance, and bioactive potential. All things considered, encapsulation of fish oil in PUL/NaCAS nanofibers offers a feasible approach to improve and safeguard the functional bioactivity of omega-rich lipids in food and medicinal systems. No standard positive control (e.g., ascorbic acid or BHT) was used; the 0% fish oil nanofibers served as the baseline for antioxidant activity.

#### 3.2.8. Antidiabetic Activity

**Figure 12:**
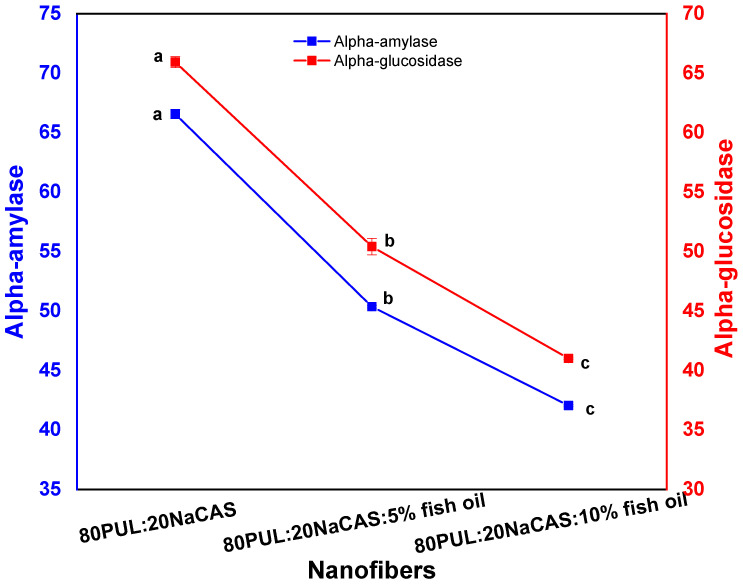
Antidiabetic activity of PUL/NaCAS nanofibers with different fish oil contents, showing α-amylase and α-glucosidase inhibition. Data are presented as mean ± SD (n = 3). Different letters indicate significant differences (*p* < 0.05).

Electrospun nanofibers’ inhibitory activities against the digestive enzymes α-amylase and α-glucosidase were also evaluated to test their potential antidiabetic activity. As shown in Figure 12 enzymatic activity significantly decreased with increasing concentrations of fish oil in the nanofiber formulations. The control formulation (80PUL:20NaCAS) showed α-amylase and α-glucosidase inhibition percentages of 65.57% and 65.94%, respectively. Incorporation of 5% and 10% fish oil led to a cumulative decrease in enzymatic activity, with the lowest inhibitory values observed for the 10% formulation (~42.05% for α-amylase and ~41.01% for α-glucosidase, Figure 12).

The results show that adding fish oil to the nanofibers potentiates their enzyme inhibition against carbohydrate-digesting enzymes. Omega-3 fatty acids, like eicosapentaenoic acid (EPA) and docosahexaenoic acid (DHA), have bioactive properties, which is the reason for this fact. The literature has already determined that these long-chain polyunsaturated fatty acids influence the metabolism of glucose, make insulin work better, and directly inhibit α-amylase and α-glucosidase from working [75].

The electrospinning technique creates fibers with ample surface areas, which helps bioactive chemicals distribute uniformly. Therefore, nanofibrous materials can enhance the availability of fish oil components and facilitate their controlled release, while the pullulan–sodium caseinate matrix enhances enzyme inhibition via synergistic polysaccharide–protein interactions, which may further amplify their therapeutic effects [76].

Using pullulan and sodium caseinate in combination as a biopolymer matrix also makes the nanofibers work better. Researchers have shown that sodium caseinate, a protein in milk, has a weak inhibitory effect on enzymes. This advantage may be due to how its peptides and proteins interact with polysaccharides [77]. Pullulan is a polysaccharide that is film-forming and serves as a barrier. It likely holds the oil in place and alters the way that it emerges [78]. All of these points indicate that electrospun pullulan/sodium caseinate nanofibers with fish oil could be useful materials for helping to keep blood sugar levels steady after eating. They would have the potential to release omega-3 fatty acids and also inhibit enzymes involved in starch breakdown. Such properties would render them a suitable choice for functional foods or nutraceuticals for type 2 diabetics.

#### 3.2.9. Oxidative Stability

**Figure 13:**
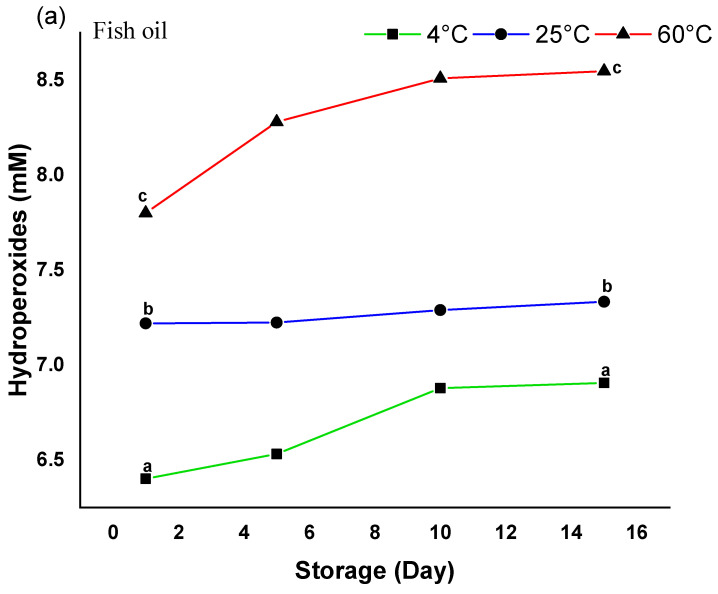

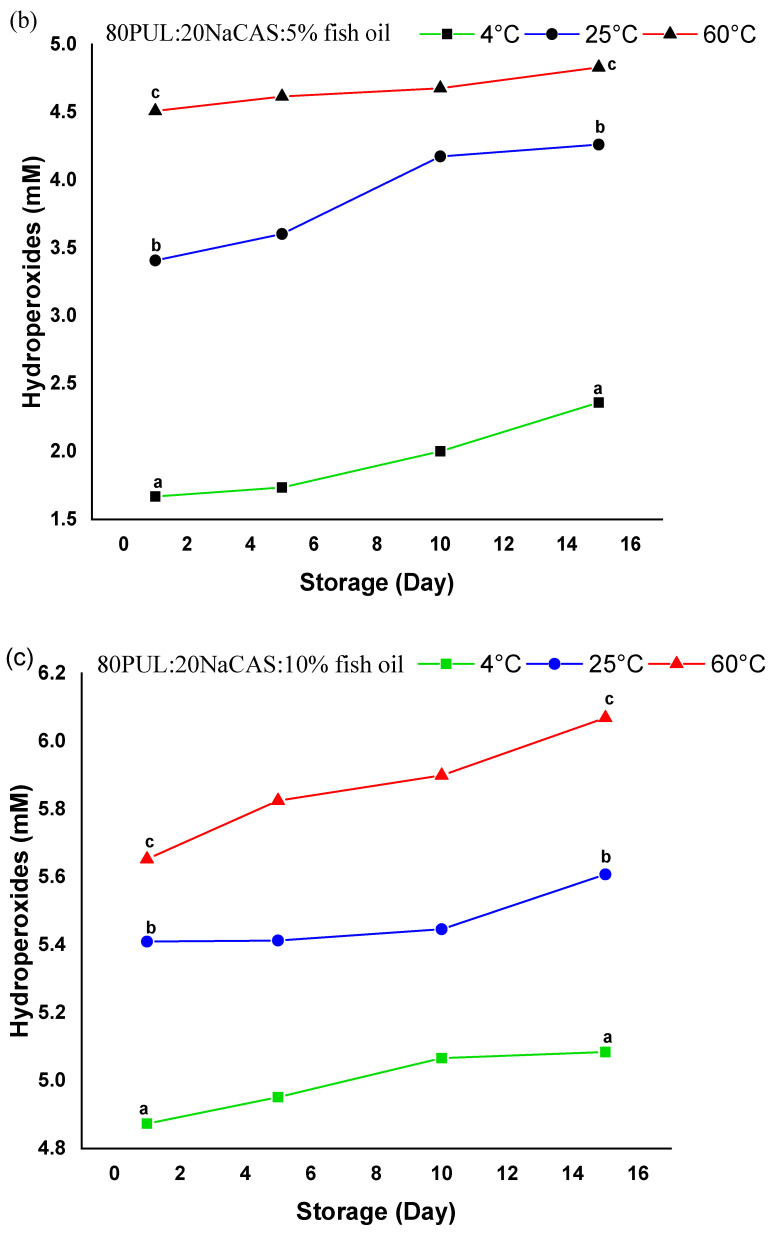
Oxidative stability of free and encapsulated fish oil in PUL/NaCAS nanofibers during 15-day storage at 4 °C, 25 °C, and 60 °C. (**a**) Fish oil, (**b**) PUL/NaCAS 5% and (**c**) PUL/NaCAS 10%. Data are mean ± SD (n = 3). Different letters (a–c) indicate significant differences (*p* < 0.05). Encapsulation, especially at 5% fish oil, significantly reduced hydroperoxide formation.

Monitoring hydroperoxide generation over a 15-day storage period at 4 °C, 25 °C, and 60 °C allowed us to assess the oxidative stability of free fish oil and encapsulated fish oil within PUL/NaCAS nanofibers (Figure 13a–c). Especially at high temperatures, free fish oil showed the fastest rise in hydroperoxide content. The peroxide value rose significantly from about 7.8 ± 0.10 to 8.6 ± 0.15 mM ^a at 60 °C (Figure 13c), therefore stressing its great sensitivity to oxidation. A consistent rise in hydroperoxides was noted even at 25 °C and 4 °C, reaching 7.3 ± 0.12 and 6.9 ± 0.09 mM ^a, respectively, by day 15.

By contrast, encapsulation greatly enhanced oxidative stability. With a final concentration of only ~4.8 ± 0.12 mM ^c at 60 °C (Figure 13a–c), the nanofiber, including 5% fish oil, had the lowest peroxide readings across all temperatures, therefore showing significant protection from oxidative destruction. Minimal oxidation (final value ~2.4 ± 0.08 mM ^c (Figure 13a–c) obtained from 4 °C storage confirms the effectiveness of the encapsulating matrix at lower temperatures. Similar trends were reported for fish oil in zein fibers, where encapsulated fish oil showed hydroperoxides lower than free fish oil [5].

Although it showed somewhat greater peroxide levels than the 5% counterpart, especially at 60 °C (up to ~6.1 ± 0.14 mM ^b Figure 13c), the nanofiber with 10% fish oil also displayed better stability relative to free oil, presumably due to structural disturbance or partial encapsulation at higher oil loading levels. This may be due to oil pooling, which led to excess oil migrating to the fiber surface and increasing oxygen exposure [50]. The slightly lower oxidative stability of the 10% formulation aligns with literature indicating that excessive oil can destabilize nanostructures and reduce protective efficiency, emphasizing the importance of optimal loading levels.

It should be noted that free fish oil mixed with PUL/NaCAS, but not electrospun, was not tested in this study. Therefore, it remains unclear whether the observed protection is due solely to the polymer matrix or whether the electrospun nanofiber structure provides additional oxidative stability. Including this control in future work would help clarify the distinct contribution of electrospinning [6].

Consistent with previous investigations on polysaccharide–protein hybrid systems [55]. These results demonstrate that encapsulation inside the pullulan/sodium caseinate nanofiber matrix provides a protective barrier against oxygen- and heat-induced lipid peroxidation. Furthermore, the better performance of the 5% fish oil formulation corresponds with the antioxidant capacity and thermal stability data (Section 3.2.9 and Section 3.2.10), achieving a balance between encapsulation efficiency, oxidative protection, and bioactive retention. These findings indicate that pullulan/sodium caseinate nanofibers have strong potential as omega-3 delivery systems in functional foods and nutraceuticals. Future studies should evaluate their performance in real food matrices, sensory attributes, and in vivo bioavailability.

#### 3.2.10. MD-Density Functional Theory Simulation (DFT)

The interaction between pullulan, sodium caseinate, and omega-3 fatty acids—specifically docosahexaenoic acid (DHA) and eicosapentaenoic acid (EPA)—was investigated using classical molecular docking and advanced density functional theory (DFT) simulations. The combined findings suggest that these components form stable, energetically favorable complexes, making them highly suitable for applications in functional foods and bioactive delivery systems.

##### Molecular Docking Analysis

**Figure 14:**
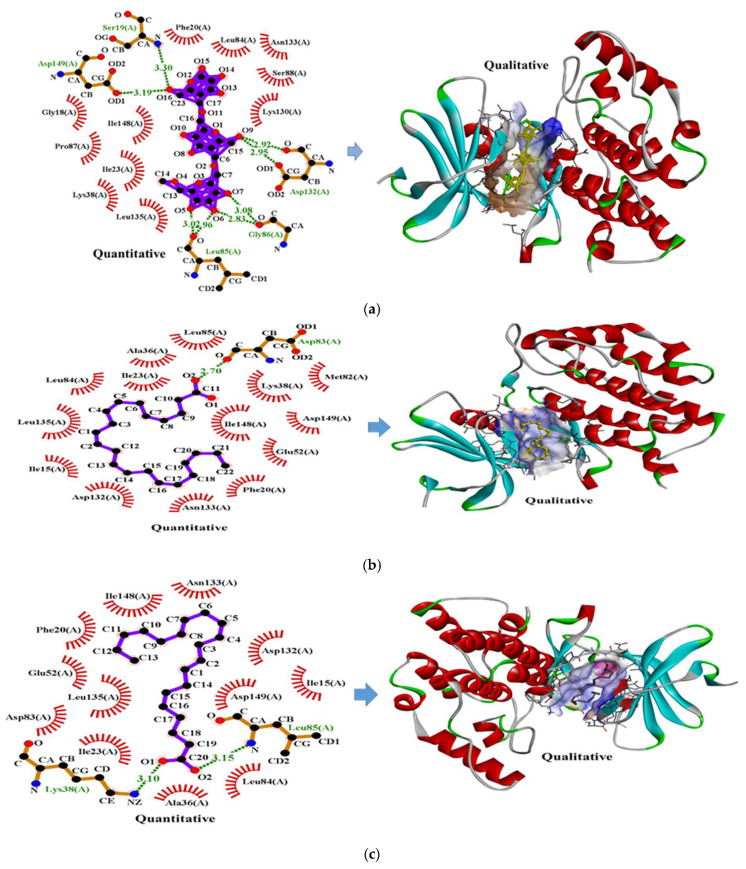
2D and 3D interaction mapping of molecular docking between pullulan, DHA, EPA, and sodium caseinate. (**a**) Pullulan–sodium caseinate interactions involve multiple hydrogen bonds (Asp149, Ser19, Asp132, Gly86, Leu85) and hydrophobic contacts stabilizing the complex. (**b**) DHA–sodium caseinate forms hydrogen bonding at Asp83 along with extensive hydrophobic interactions, while (**c**) EPA–sodium caseinate exhibits hydrogen bonds at Leu85 and Lys38 with additional hydrophobic contacts. These interactions highlight the cooperative role of hydrogen bonding and hydrophobic forces in stabilizing the polysaccharide–protein and lipid–protein complexes.

Molecular docking analysis revealed that both pullulan and omega-3 fatty acids exhibit strong and thermodynamically favorable interactions with sodium caseinate (Figure 14a–c). The pullulan–sodium caseinate complex displayed a binding energy of −7.3 kcal/mol (Figure 13a), stabilized by eight hydrogen bonds and eleven hydrophobic interactions. Key interacting residues such as Asp132, Gly86, and Leu85 were involved in forming multiple hydrogen bonds with hydroxyl groups on the pullulan backbone. These interactions underscore the cooperative role of hydrogen bonding and hydrophobic forces in stabilizing protein–polysaccharide conjugates, in line with prior studies on such complexation mechanisms [79,80].

The DHA–casein and EPA–casein complexes showed binding energies of −6.2 kcal/mol and −6.0 kcal/mol, respectively (Figure 14b,c), indicating thermodynamically favorable interactions. In the DHA–casein complex, one hydrogen bond was observed involving Asp83, along with fourteen hydrophobic interactions distributed across the binding interface (Figure 14b). For the EPA–casein system, two hydrogen bonds were formed with Leu85 and Lys38, accompanied by twelve hydrophobic interactions contributing to the overall stabilization (Figure 14c). These data suggest that while hydrogen bonding plays a specific role in anchoring the molecules, hydrophobic interactions are the dominant force maintaining the structural integrity of these lipid–protein complexes. Such interactions support efficient encapsulation of omega-3 fatty acids, potentially enhancing antioxidant activity and sustained enzyme inhibition, which aligns with reported mechanisms for protein-based delivery of lipophilic nutrients [81,82].

##### Frontier Molecular Orbital Analysis and Electronic Stability

**Figure 15:**
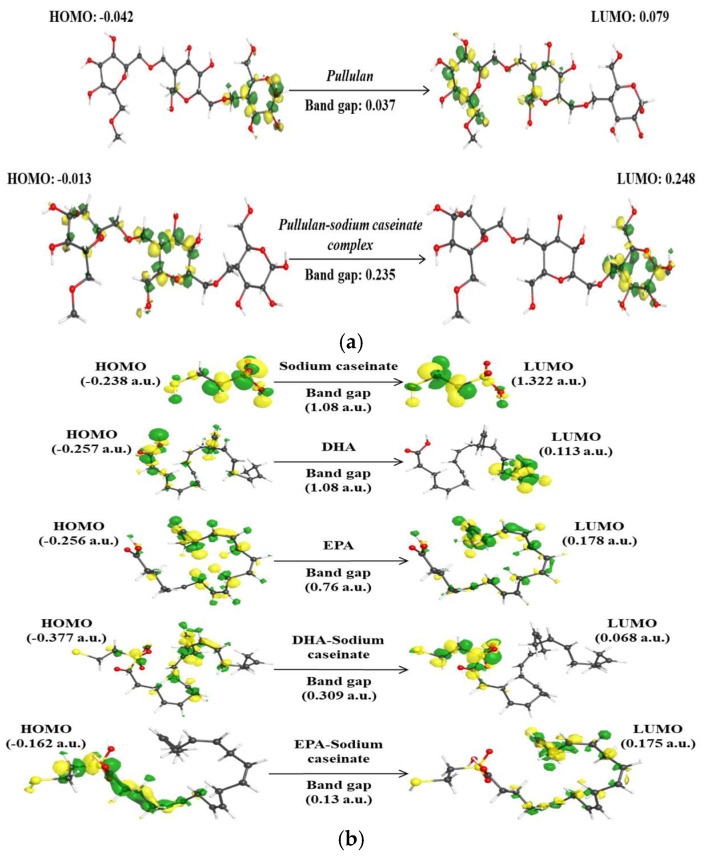
Chemical stability based on HOMO–LUMO band gap analysis: (**a**) Pullulan–sodium caseinate complex showing increased band gap compared to pure pullulan; (**b**) DHA– and EPA–caseinate systems showing similar band gap expansion trends.

DFT simulations offered comprehensive insight into the electronic structure and stability of the studied complexes. Notably, the HOMO–LUMO band gap of pullulan increased from 0.037 eV to 0.235 eV upon interaction with sodium caseinate (Figure 15a), signifying enhanced chemical stability and reduced molecular reactivity [83]. A similar trend of band gap expansion was observed in DHA– and EPA–caseinate systems (Figure 15a,b), highlighting their increased resistance to electron excitation.

##### Atomic Energy Distribution as a Basis for Thermodynamic Assessment

**Figure 16:**
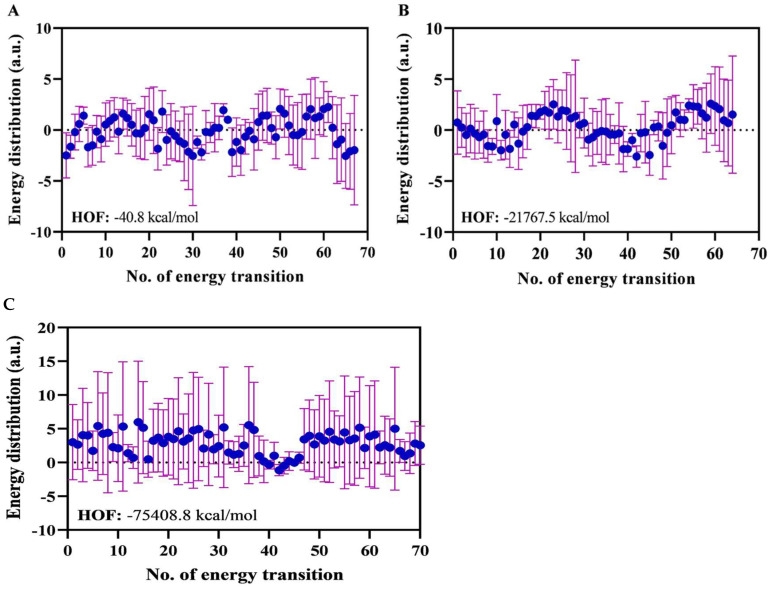
Atomic energy distribution analysis for DHA (**A**), EPA (**B**), and pullulan complexed individually with sodium caseinate (**C**) over a 200 ns simulation time frame following DFT analysis. The heat of formation (HOF) and energy transitions indicate energetically favorable and stable interactions, with final energies of −1567.2 a.u. for DHA–sodium caseinate, −1419.8 a.u. for EPA–sodium caseinate, and −162,232.5 a.u. for pullulan–sodium caseinate. These results highlight the thermodynamic stability and compatibility of the lipid–protein and polysaccharide–protein complexes.

Thermodynamic evaluation based on atomic energy distribution (Figure 16A for DHA, B EPA, C for Pullulan) demonstrated favorable heat of formation (HOF) values: −162,232.5 a.u. for the pullulan–caseinate complex, −1567.2 a.u. for DHA–caseinate, and −1419.8 a.u. for EPA–caseinate (Figure 16A–C). These negative values confirm that the systems are energetically stable and support their molecular compatibility. Among the lipid–protein systems, this stability aligns with the lower peroxide values and improved oxidative protection observed in the 5% fish oil nanofibers, linking computational predictions to experimental outcomes. The DHA–caseinate complex exhibited superior thermodynamic stability, likely due to DHA’s extended carbon chain and higher degree of unsaturation, which promote enhanced van der Waals and hydrophobic interactions with the casein matrix. The atomic energy distribution further reveals a more uniform energy transition pattern for DHA, indicating improved conformational equilibrium. This higher thermodynamic efficiency aligns with other computational metrics such as SCF stability and HOMO–LUMO band gap, collectively reinforcing the robustness of the DHA–caseinate system for encapsulation and oxidative protection of omega-3 fatty acids [79].

Overall, the combination of widened HOMO–LUMO gaps, localized FMO charge regions, and sustained SCF energy stability illustrates the effectiveness of pullulan–sodium caseinate systems as functional delivery matrices. These computational predictions align with the observed in vitro results, where the 5% fish oil nanofibers showed lower peroxide values, higher antioxidant activity, and enhanced α-amylase and α-glucosidase inhibition, confirming that the stable molecular interactions improve bioactive retention and functionality. These properties support their use in advanced food hydrocolloid applications aimed at preserving the quality and bioactivity of polyunsaturated lipids in fortified products.

## 4. Conclusions

An effective approach to enhance the oxidative stability of fish oil is its encapsulation within pullulan/sodium caseinate nanofibers using electrospinning. Incorporating 5% fish oil yields optimal outcomes according to evaluations of structural integrity, thermal resistance, and functionality, including enhanced fiber strength, increased hydrophobicity, elevated antioxidant activity (DPPH ~33%, ABTS 46 mg TE/g, TPC 115 mg GAE/g), and markedly reduced lipid degradation during storage (hydroperoxide values decreased from 8.6 to 4.8 mM after 15 days at 60 °C). Despite the 10% oil formulation demonstrating superior loading capacity, it exhibited structural integrity issues and reduced bioactive efficacy. The consistency of fiber morphology, emulsion stability, matrix interactions, and bioactivity underscores the critical importance of optimizing the oil-to-polymer ratio. These findings highlight the potential of PUL/NaCAS nanofibers as a scalable and protective delivery platform for functional lipids in food and nutraceutical applications. Future studies should explore higher oil loadings, long-term stability, sensory evaluation, in vivo bioactivity, and industrial-scale processing to fully realize the practical applicability of this system. Limitations: This study was limited to 0–10% oil, in vitro assays, short-term storage, and lab-scale electrospinning, with molecular interactions inferred from simulations. Future studies should include sensory evaluation and longer-term storage (1–3 months) to link oxidation with perceptible changes and assess shelf-life, along with higher oil loading, in vivo bioactivity, and scalability.

## Data Availability

The original contributions presented in this study are included in the article. Further inquiries can be directed to the corresponding author.
